# Serum Albumin and Albumin-Based Nutritional-Inflammatory Indices Across the 2026 ESC Heart Failure Phenotypes: Associations with Recorded All-Cause Mortality

**DOI:** 10.3390/jcm15218231

**Published:** 2026-10-22

**Authors:** Bozhidar Krastev, Natalia Spasova, Rumena Savova, Angelina Borizanova, Radostina Ilieva, Slavi Yakov, Elena Kinova, Assen Goudev

**Affiliations:** 1 Department of Emergency Medicine, Medical University-Sofia, 1527 Sofia, Bulgaria; spasova.natalia@gmail.com (N.S.); rumenasavova@gmail.com (R.S.); borizanowa@abv.bg (A.B.); radostilieva@yahoo.com (R.I.); slavi_yakov93@abv.bg (S.Y.); kinova.e@abv.bg (E.K.); agoudev@abv.bg (A.G.); 2 Cardiology Department, University Hospital “Tsaritsa Yoanna-ISUL”, 1527 Sofia, Bulgaria

**Keywords:** heart failure, serum albumin, recorded all-cause mortality, NT-proBNP, nutritional-inflammatory markers

## Abstract

**Background**: Serum albumin reflects nutritional status, inflammation, congestion, and organ dysfunction in heart failure (HF). We examined its association with recorded all-cause mortality and compared it with the prognostic nutritional index (PNI) and hemoglobin–albumin–lymphocyte–platelet (HALP) index. **Methods**: This retrospective single-center study included 492 patients hospitalized with decompensated HF with available serum albumin, left ventricular ejection fraction (LVEF), and N-terminal pro-B-type natriuretic peptide (NT-proBNP), classified as heart failure with reduced ejection fraction (HFrEF; LVEF < 50%) or heart failure with preserved ejection fraction (HFpEF; LVEF ≥ 50%) using the 2026 European Society of Cardiology (ESC) framework. Exact death dates were unavailable, and potential observation time varied between patients. Logistic regression evaluated each marker separately after adjustment for clinical characteristics, NT-proBNP, and renal function. **Results**: There were 177 recorded deaths. Lower albumin (odds ratio [OR] 1.60 per 5 g/L decrease, 95% confidence interval [CI] 1.30–1.96), PNI (OR 1.43 per 5-unit decrease, 95% CI 1.21–1.67), and HALP (OR 1.15 per 10-unit decrease, 95% CI 1.04–1.27) were associated with mortality. Adding albumin to the clinical + NT-proBNP model improved model fit (likelihood-ratio *p* < 0.001) and increased apparent area under the receiver operating characteristic curve (AUC) from 0.689 to 0.731; PNI and HALP yielded AUCs of 0.730 and 0.710. For the primary albumin model, bootstrap optimism correction reduced the AUC from 0.735 to 0.702, with a corrected calibration slope of 0.822. Associations were similar in the sensitivity analysis restricted to at least 12 months of potential observation. Interaction tests provided no evidence that the associations differed by HF phenotype, but did not establish equivalence. **Conclusions**: Lower serum albumin was associated with recorded mortality and added information beyond clinical variables and NT-proBNP. Comparisons with PNI and HALP remain exploratory. Clinical use requires external validation and follow-up with known event dates.

## 1. Introduction

Heart failure (HF) affects more than 60 million people worldwide and becomes more prevalent with age. Population aging and improved survival after cardiovascular disease have increased the number of people living with HF. Despite advances in treatment, recurrent hospitalization, impaired quality of life, and substantial mortality remain common [1,2].

Obesity increases the risk of developing HF, yet observational studies in established HF have reported better survival among overweight and moderately obese patients—the obesity paradox [3]. This observation does not demonstrate a protective effect of obesity. Differences in age and disease severity may explain part of the association [4].

Inflammatory mechanisms are implicated in HF progression. They have been linked to endothelial dysfunction, myocardial fibrosis and remodeling, and renal and other organ involvement. Inflammation may also reflect the severity of established HF [5,6]. The ATHENA and HERMES trials were designed to investigate anti-inflammatory treatment in HF [7].

Inflammation may reduce albumin synthesis and increase its catabolism and capillary leakage; hepatic congestion, impaired liver function, renal protein loss, and haemodilution may also lower circulating concentrations [8,9]. Albumin contributes to plasma oncotic pressure and has transport, antioxidant, and anti-inflammatory properties. A low concentration may therefore reflect nutritional impairment, inflammation, congestion, and organ dysfunction rather than malnutrition alone.

A systematic review and meta-analysis including more than 44,000 patients associated hypoalbuminemia with in-hospital and long-term mortality in HF [10]. Albumin is inexpensive and routinely measured, but its added value beyond clinical characteristics and N-terminal pro-B-type natriuretic peptide (NT-proBNP) requires evaluation.

The prognostic nutritional index (PNI), which combines albumin with lymphocyte count, has been associated with adverse outcomes in HF [11]. The hemoglobin–albumin–lymphocyte–platelet (HALP) index additionally incorporates hemoglobin and platelet count and has also been associated with mortality [12]. The C-reactive protein-to-albumin ratio (CAR) combines inflammatory and albumin-related information and has been associated with mortality in HF [13]. Whether composite indices offer an advantage over albumin alone remains uncertain.

We evaluated the association between serum albumin and recorded all-cause mortality in patients hospitalized with HF and examined whether this association differed between heart failure with reduced ejection fraction (HFrEF) and heart failure with preserved ejection fraction (HFpEF) under the 2026 European Society of Cardiology (ESC) phenotype framework [14]. We compared the apparent performance of separate albumin, PNI, and HALP models and assessed the added model fit of each marker beyond clinical variables and NT-proBNP.

## 2. Materials and Methods

### 2.1. Study Design and Population

We reviewed hospital records of patients admitted for worsening or decompensated chronic heart failure to the Cardiology Clinic at University Hospital “Tsaritsa Yoanna–ISUL”, Sofia, Bulgaria, from September 2023 to October 2025.

Dyspnoea and fatigue were the most frequently documented symptoms at presentation.

The diagnosis was made by the treating cardiology team on the basis of symptoms, examination findings, laboratory tests including NT-proBNP, and echocardiography.

Diuretics were given during the index admission according to the patient’s clinical needs.

We relied on routine records for diagnoses and treatment, without additional adjudication for this analysis.

For patients with repeated eligible hospitalizations, the first eligible admission was used.

The source cohort comprised 1284 unique patients hospitalized with exacerbated or decompensated heart failure. Of these, 345 were excluded because of active infection, active malignancy, autoimmune disease, or systemic corticosteroid treatment. Among the remaining 939 patients, NT-proBNP measurements were available for 637; the other 302 were excluded. Of these 637 patients, 492 also had available serum albumin measurements and were included in the study cohort. The remaining 145 were excluded because albumin measurements were unavailable. Left ventricular ejection fraction (LVEF) was available for all 492 included patients.

NT-proBNP and albumin were measured in routine care rather than under a study-specific testing protocol; no NT-proBNP concentration threshold was used for inclusion. Comparable baseline data for patients outside the included cohort were not systematically available.

Mortality status was available for 491 patients. The patient with missing mortality status was retained in the descriptive analyses but excluded from mortality analyses. Blood-count measurements were missing for a different patient, so PNI and HALP could be calculated in 491 patients, of whom 490 had known mortality status. The source population for this study was also used in our previously published analysis of the systemic immune-inflammation index (SII) in heart failure [15]. The earlier study focused on SII, whereas the present work examines serum albumin, PNI, and HALP. The present analytical cohort was defined by available albumin, LVEF, and NT-proBNP measurements, and mortality status was ascertained on 17 September 2026.

Clinical data extracted from the hospital records included age, sex, cardiovascular comorbidities, LVEF, and pre-admission treatment. Laboratory variables included NT-proBNP, renal function, hemoglobin, neutrophil, lymphocyte, and platelet counts, C-reactive protein (CRP), high-sensitivity cardiac troponin I, and serum albumin. Cardiac troponin I was measured using a high-sensitivity assay from Abbott Diagnostics. Obesity was analyzed as the binary variable recorded in the clinical dataset, without a new body mass index (BMI)-based classification. The earliest available laboratory results during the index hospitalization were used. The albumin assay method and analyzer were not recorded in the analysis dataset. Samples were generally obtained at or shortly after admission, but their timing relative to treatment initiation or intensification could not be standardized retrospectively. Missing values were not imputed; each model used patients with complete data for its variables.

Estimated glomerular filtration rate (eGFR) was based on the Modification of Diet in Renal Disease (MDRD) equation used in routine laboratory reporting.

The patient selection process is shown in Supplementary Figure S2.

### 2.2. Heart Failure Phenotypes

LVEF was used to subclassify patients with clinically diagnosed HF according to the 2026 ESC phenotype framework applied in this analysis [14]. HFrEF was defined as LVEF < 50% and HFpEF as LVEF ≥ 50%. Patients previously classified as having mildly reduced ejection fraction were therefore included within the HFrEF group.

### 2.3. Serum Albumin and Nutritional-Inflammatory Indices

Serum albumin was the primary laboratory marker of interest and was analyzed as a continuous variable. Effect estimates were expressed per 5 g/L decrease in albumin.

Two routinely derived nutritional-inflammatory indices were evaluated as secondary markers. The prognostic nutritional index (PNI) was calculated as

PNI = albumin (g/L) + 5 × lymphocyte count (10^9^/L).

The hemoglobin–albumin–lymphocyte–platelet index (HALP) was calculated as

HALP = hemoglobin (g/L) × albumin (g/L) × lymphocyte count (10^9^/L)/platelet count (10^9^/L).

PNI was analyzed per 5-unit decrease and HALP per 10-unit decrease.

### 2.4. Outcome Definition

Recorded all-cause mortality was the study outcome. Vital status was checked on 17 September 2026 using the National Health Insurance Fund database together with the hospital’s electronic medical records.

Exact dates of death and dates of last known contact were not available. The available mortality-status data did not allow us to determine whether deaths occurred within the first 12 months after admission.

Mortality was therefore analyzed as a binary outcome rather than using time-to-event methods. Accordingly, the reported odds ratios (ORs) represent associations with recorded mortality status and should not be interpreted as hazard ratios or as estimates of mortality risk over a fixed follow-up period.

Mortality status was available for 491 of the 492 patients. Potential observation time was defined as the interval from the index hospitalization date to 17 September 2026, irrespective of recorded vital status; it does not represent observed survival time.

### 2.5. Statistical Analysis

Continuous variables are presented as median [interquartile range (IQR)], and categorical variables as number and percentage. Comparisons between HFrEF and HFpEF were performed using the Mann–Whitney U test for continuous variables and the chi-square or Fisher’s exact test for categorical variables, as appropriate.

We analyzed recorded mortality using multivariable logistic regression. The clinical adjustment set comprised age, sex, HF phenotype, coronary artery disease, previous myocardial infarction, previous stroke, diabetes mellitus, hypertension, obesity, atrial fibrillation, and dyslipidemia.

Age was entered as a continuous variable and reported per 10-year increase. HFpEF was used as the reference phenotype. All covariates were entered simultaneously; no stepwise selection procedure or univariable *p*-value screening was used.

Models were fitted by unpenalized maximum likelihood using a custom Newton–Raphson implementation with step halving. Predictors were standardized for numerical fitting, and coefficients and standard errors were transformed back to the reported scales. Standard errors were obtained from the inverse observed information matrix. Confidence intervals (CIs) and two-sided *p*-values for individual coefficients used the Wald normal approximation.

Log_2_-transformed NT-proBNP was used in regression models, with odds ratios expressed per doubling of NT-proBNP. eGFR was entered per 10 mL/min/1.73 m^2^ increase.

Each model included the clinical covariates, log_2_-transformed NT-proBNP, eGFR, and one marker. Albumin, PNI, and HALP were analyzed separately because both indices contain albumin. The primary albumin model included 491 patients, whereas the PNI and HALP models each included 490 because one patient lacked the blood-count measurements required to calculate these indices.

#### 2.5.1. Incremental Model Performance

Incremental discrimination was evaluated using nested models on a common complete-case population. The first model contained the clinical covariates alone. NT-proBNP was then added, followed by albumin, PNI, or HALP in separate models. These comparisons addressed added information beyond clinical characteristics and NT-proBNP. Renal adjustment was addressed in the fully adjusted association models; eGFR was not included in the sequential models.

Model discrimination was quantified by the area under the receiver operating characteristic (ROC) curve (AUC), calculated from model-predicted probabilities. Likelihood-ratio (LR) tests were the primary tests of incremental model fit. Changes in AUC were reported with approximate 95% confidence intervals based on the DeLong covariance estimate. These intervals describe apparent discrimination in the development sample. Because the models were nested and fitted in the same patients, DeLong hypothesis tests were not used as the primary evidence for incremental value [16]. Overall predictive accuracy was assessed with the Brier score.

#### 2.5.2. Calibration and Internal Validation

For the primary albumin model, calibration was examined using the Hosmer–Lemeshow test across 10 groups based on predicted-probability deciles (8 degrees of freedom [df]).

Calibration intercept and slope were estimated jointly by logistic regression of mortality status on the model linear predictor.

Internal validation of the primary albumin model used 1000 valid bootstrap resamples from 1001 draws (random seed 20260929). Fits that failed to converge or produced a singular or severely ill-conditioned information matrix (condition number >10^13^) were excluded and redrawn; the single excluded draw showed quasi-complete separation. In each valid resample, the model was refitted and evaluated in both the bootstrap sample and the original cohort. For each performance measure, mean optimism was the bootstrap-sample estimate minus the original-cohort estimate for the same refitted model. Mean optimism was subtracted from the apparent AUC, Brier score, calibration slope, and calibration intercept. Bootstrap validation was limited to the primary albumin model.

#### 2.5.3. Multicollinearity

Multicollinearity was examined using variance inflation factors and tolerance values. Because albumin, PNI, and HALP were analyzed in separate models, multicollinearity diagnostics were calculated separately for each model.

#### 2.5.4. Interaction Analysis

For each marker, we assessed whether the mortality association varied by LVEF phenotype by adding a marker-by-phenotype interaction term to the adjusted model. A likelihood-ratio test compared the models with and without this term.

#### 2.5.5. Restricted Cubic Spline Analysis

We used a restricted cubic spline for albumin in the fully adjusted mortality model to examine departures from a linear association on the log-odds scale.

Four knots were placed at the 5th, 35th, 65th, and 95th percentiles of the albumin distribution in the 491 complete cases used for this analysis (28.0, 36.5, 41.0, and 46.0 g/L, respectively), with the median albumin value (39.0 g/L) used as the reference. Likelihood-ratio tests compared the spline model with a model omitting albumin (overall association) and with a model containing a linear albumin term (non-linearity).

#### 2.5.6. Sensitivity Analyses

Patients entered the cohort at different dates and had different potential periods during which mortality could be recorded. Potential observation time was summarized in months as the number of days from the index hospitalization to 17 September 2026 divided by 30.4375. For the sensitivity analysis, eligibility was defined by the calendar cutoff of index hospitalization on or before 17 September 2025, at least one calendar year before outcome ascertainment.

Index dates were available for all 492 patients. In the subgroup with at least 12 months of potential observation, recorded all-cause mortality was analyzed using multivariable logistic regression with the original covariates and marker scales. Each marker model used its own complete-case sample. This restriction did not equalize observation time and does not substitute for time-to-event analysis.

An exploratory sensitivity analysis additionally included log_2_-transformed CRP in each of the albumin, PNI, and HALP models, retaining the original covariates and marker scales. Analyses used patients with complete data for all model variables. The corresponding models without CRP were refitted in the same patients to distinguish the effect of CRP adjustment from changes in the analysis sample.

All tests were two-sided, with *p* < 0.05 considered statistically significant. Analyses were performed in Python 3.12.14 using NumPy 2.3.5, pandas 2.2.3, SciPy 1.17.0, and scikit-learn 1.8.0. The secondary marker analyses were interpreted as exploratory.

### 2.6. Ethical Considerations

The study was conducted in accordance with the Declaration of Helsinki and was approved by the Institutional Ethics Committee of University Hospital “Tsaritsa Yoanna–ISUL”, Sofia, Bulgaria (Approval No. 14/2004RS; 20 April 2026). Because of the retrospective design and the use of anonymized routinely collected clinical data, the requirement for written informed consent was waived.

## 3. Results

Among 492 patients, 215 (43.7%) had HFrEF and 277 (56.3%) had HFpEF (Table 1). Patients with HFpEF were slightly older, whereas men were more frequent in HFrEF. HFrEF was associated with higher NT-proBNP, creatinine, and high-sensitivity cardiac troponin I, and with more frequent previous myocardial infarction and dyslipidemia. Albumin, PNI, and HALP did not differ significantly between the phenotypes. Mortality status was known in 491 patients, with 177 deaths (36.0%). Potential observation time ranged from 11.6 to 35.6 months, with a median of 21.9 months (IQR 17.2–27.0).

In the multivariable clinical model (Table 2), increasing age was associated with higher odds of recorded all-cause mortality (OR 1.32 per 10-year increase, 95% CI 1.08–1.60, *p* = 0.005). Previous myocardial infarction (OR 2.34, 95% CI 1.24–4.42, *p* = 0.009) and atrial fibrillation (OR 1.63, 95% CI 1.09–2.45, *p* = 0.017) were also associated with higher odds of recorded mortality. HFrEF phenotype was not significantly associated with the outcome after adjustment (OR 1.30, 95% CI 0.87–1.95, *p* = 0.198). The overall model was significant (LR χ^2^ = 43.35, *p* < 0.001), with an AUC of 0.663.

In adjusted analyses (Table 3), lower serum albumin, PNI, and HALP were each significantly associated with higher odds of recorded all-cause mortality. A 5 g/L decrease in albumin was associated with a 60% increase in the odds of recorded mortality (OR 1.60, 95% CI 1.30–1.96, *p* < 0.001). Similarly, a 5-unit decrease in PNI was associated with higher odds of recorded mortality (OR 1.43, 95% CI 1.21–1.67, *p* < 0.001), while a 10-unit decrease in HALP was also significant (OR 1.15, 95% CI 1.04–1.27, *p* = 0.005). The corresponding AUCs were 0.735 for albumin, 0.732 for PNI, and 0.710 for HALP, with significant incremental likelihood-ratio tests for all three markers.

Calibration and internal validation of the primary albumin model are presented in Supplementary Table S1. The model included 491 patients, with 177 recorded deaths. The apparent AUC was 0.735, and the Brier score was 0.194. The Hosmer–Lemeshow test did not indicate evidence of poor fit (χ^2^ = 11.67, *p* = 0.167). After 1000 valid bootstrap resamples, the optimism-corrected AUC was 0.702, with an optimism-corrected Brier score of 0.207 and a calibration slope of 0.822. These validation results apply only to the primary albumin model.

**Figure 1:**
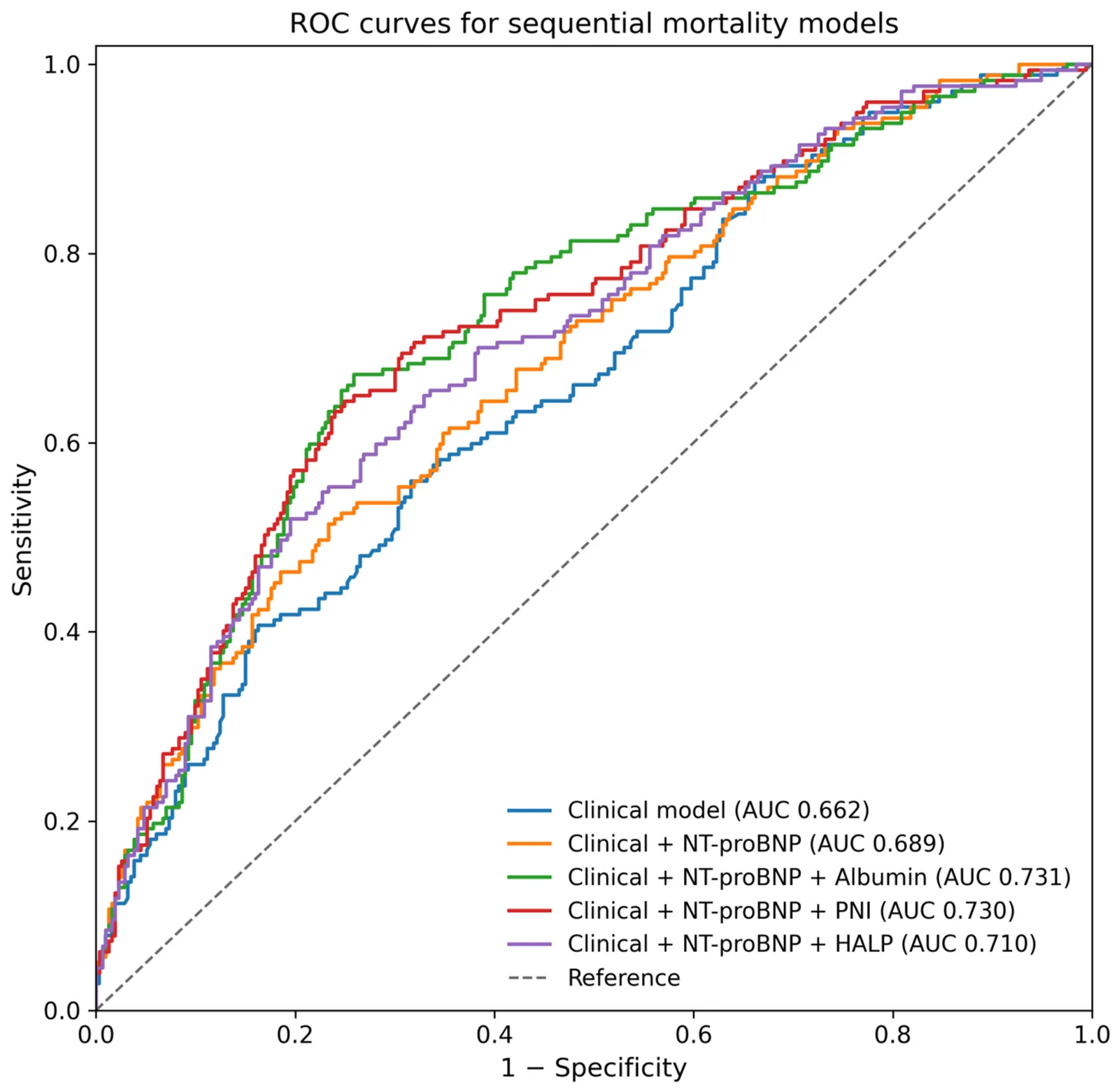
ROC curves for sequential models of recorded all-cause mortality. ROC curves are shown for the clinical model, the clinical model with NT-proBNP, and the latter model after separate addition of albumin, PNI, or HALP. All curves are based on the same 490 patients. Abbreviations. ROC: Receiver operating characteristic; AUC: area under the receiver operating characteristic curve; NT-proBNP: N-terminal pro-B-type natriuretic peptide; PNI: Prognostic nutritional index; HALP: Hemoglobin–albumin–lymphocyte–platelet index.

Sequential models are presented in Table 4 and Figure 1. Unlike the fully adjusted association models in Table 3, these models omitted eGFR and used the same 490 patients throughout. Apparent AUC increased from 0.662 for the clinical model to 0.689 after adding NT-proBNP. Adding albumin increased AUC to 0.731 (change in AUC [ΔAUC] 0.042, 95% CI 0.012–0.071; LR χ^2^ = 20.35, *p* < 0.001). PNI yielded AUC 0.730 (ΔAUC 0.041, 95% CI 0.012–0.069; LR χ^2^ = 19.66, *p* < 0.001), and HALP yielded AUC 0.710 (ΔAUC 0.021, 95% CI 0.001–0.041; LR χ^2^ = 8.79, *p* = 0.003). These comparisons assess each marker against the clinical + NT-proBNP model and do not establish superiority of one marker over another.

No evidence of relevant multicollinearity was observed in any of the adjusted models, with all variance inflation factors ≤1.54 and tolerance values ≥0.649 (Supplementary Table S2).

Although the adjusted associations appeared numerically stronger in HFrEF, there was no statistically significant evidence that the associations of albumin, PNI, or HALP with recorded mortality differed by LVEF phenotype (*p* for interaction = 0.212, 0.166, and 0.275, respectively; Supplementary Table S3).

Restricted cubic spline analysis showed a significant overall association between serum albumin and recorded all-cause mortality (LR χ^2^ = 24.56, df = 3; *p* < 0.001), with no evidence of significant non-linearity (LR χ^2^ = 3.46, df = 2; *p* = 0.177). The spline model had an AUC of 0.739 and a Brier score of 0.193. These findings were compatible with the linear specification used in the primary analysis (Supplementary Table S5 and Figure S1).

Restricting the analysis to at least 12 months of potential observation retained 482 patients for albumin and 481 for PNI/HALP, with 173 deaths in each model. The associations were similar to the primary results: albumin OR 1.66 (95% CI 1.34–2.04), PNI OR 1.45 (95% CI 1.23–1.70), and HALP OR 1.15 (95% CI 1.05–1.27) at their original scales (Supplementary Table S4). This restriction excluded only nine patients from the primary albumin model.

After additional adjustment for CRP, albumin and PNI remained associated with recorded all-cause mortality. The association for HALP was attenuated and was no longer statistically significant (*p* = 0.066; Supplementary Table S6).

## 4. Discussion

Among the patients in this albumin analysis, lower serum albumin concentrations were associated with recorded mortality after accounting for clinical characteristics, NT-proBNP, and kidney function.

The association was similar when the analysis was restricted to patients with at least 12 months of potential observation. Adding albumin improved model fit and apparent discrimination beyond clinical variables and NT-proBNP. PNI performed similarly, whereas HALP showed a smaller gain in apparent discrimination.

The primary albumin model had an apparent AUC of 0.735 and a bootstrap optimism-corrected AUC of 0.702, with a corrected calibration slope of 0.822. PNI and HALP were not internally validated, so comparisons based on their apparent AUCs remain exploratory. These findings do not establish clinical utility, and none of the models has been externally validated.

The albumin finding is consistent with earlier HF studies. Uthamalingam et al. reported higher 1-year mortality in hospitalized patients with acute decompensated HF and hypoalbuminemia [17]. Similar associations were reported in advanced systolic HF by Horwich et al. [18] and in HFpEF by Liu et al. [19]. A meta-analysis of more than 44,000 patients subsequently linked hypoalbuminemia to in-hospital and longer-term mortality [10].

Low albumin in HF can reflect several processes occurring together. Inflammation may suppress hepatic synthesis and increase vascular leakage, while congestion can impair intestinal nutrient absorption and contribute to hepatic dysfunction. Reduced dietary intake, renal or intestinal protein loss, and dilution from fluid retention may also lower albumin concentrations [8]. These mechanisms overlap: congestion may worsen nutritional status and promote inflammation, while inflammation can further alter albumin metabolism. The association observed in our cohort may therefore reflect their combined effects rather than malnutrition alone. The available data did not allow us to distinguish their individual contributions.

In ALBIMED-HF, Barbiero et al. studied 498 older patients hospitalized with acute HF and found that hypoalbuminemia remained associated with 12-month mortality after multivariable adjustment [20]. That cohort was considerably older than ours.

A 2025 systematic review included 91 studies; its pooled prevalence analysis comprised 90 studies with 51,086 patients. Malnutrition prevalence was 44.6% overall and 49.3% among hospitalized patients. Malnutrition was associated with approximately twice the mortality risk, although heterogeneity was substantial and certainty of evidence was very low [21]. These markers do not replace a nutritional assessment.

For PNI, Cheng et al. reported associations with all-cause and cardiovascular mortality in acute HF [22], and a meta-analysis of 12 studies and more than 9000 patients supported its association with adverse long-term outcomes [11]. In our cohort, the PNI model did not show a numerical advantage over the albumin model in apparent discrimination. The analysis was not designed to establish equivalence between the markers.

Solano et al. examined PNI in PARADIGM-HF and PARAGON-HF [23]. Lower PNI identified patients with greater frailty, comorbidity, and congestion, and was associated with worse outcomes after adjustment in both trial populations. In our cohort, the interaction tests did not provide evidence that the associations of albumin, PNI, or HALP with recorded mortality differed between HFrEF and HFpEF (Supplementary Table S3). This does not establish equivalence. The subgroup estimates should be interpreted cautiously given the uncertainty reflected in their confidence intervals, and differences between phenotypes cannot be ruled out.

Liu et al. linked lower HALP to short- and long-term mortality in 730 hospitalized patients with HF [12]. Zhen et al. subsequently reported associations in 4286 patients with HF admitted to intensive care in the Medical Information Mart for Intensive Care IV (MIMIC-IV) database, with relationships that were not uniformly linear [24]. In our cohort, HALP was associated with mortality in the primary model and yielded a smaller gain in apparent discrimination than albumin or PNI.

Albumin, PNI, and HALP did not differ significantly between HFrEF and HFpEF at baseline. Evidence for albumin in HFpEF also comes from a 2025 meta-analysis of 20 studies involving more than 30,000 patients; the albumin analysis included 16 studies and linked higher concentrations to fewer adverse outcomes [25]. Comparisons with earlier studies should account for differences in populations, endpoints, and ejection fraction definitions.

PNI combines albumin with lymphocyte count to provide information about nutritional and immune status. In HF, lymphopenia may accompany malnutrition, inflammation, and neurohormonal activation [12,22]. HALP also includes hemoglobin, which reflects anemia and oxygen-carrying capacity, and platelet count, given the role of platelets in inflammation and thrombosis [12]. However, platelet count does not directly measure platelet activation. Because these components are influenced by several processes, neither index is a specific measure of nutrition or inflammation. We analyzed PNI and HALP as composite markers and did not assess the contribution of each component. Whether either index improves prediction over albumin alone remains to be tested in external cohorts.

The CRP-adjusted analysis supported the findings for albumin and PNI, while the association for HALP weakened. Adjustment for CRP alone does not establish that these associations are independent of inflammation.

Albumin may complement NT-proBNP and clinical assessment, but these observational associations do not show that low albumin causes poor outcomes or that increasing its concentration would improve prognosis [8].

The retrospective, single-center design limits generalizability and permits residual confounding. Selection depended partly on the availability of routine laboratory data; exclusion counts by clinical criterion and comparable data for non-included patients were unavailable. The clinical exclusions also limit applicability to patients with the excluded conditions. Sampling relative to treatment was not standardized, and single measurements may reflect transient congestion or haemodilution. Albumin assay details were not recorded in the analysis dataset. CRP was included only in an exploratory sensitivity analysis restricted to patients with complete data. Treatment and direct measures of congestion were not included in the models.

Exact death dates were unavailable, so the outcome represents recorded vital status at a common ascertainment date with unequal potential observation periods. The calendar-year restriction removed only nine patients from the albumin model and did not equalize observation time. With 177 deaths and 14 predictor parameters in the primary linear models, overfitting remains possible. Non-linearity was assessed for albumin; the other continuous predictors were assumed to have linear effects on the log odds after their specified transformations, without separate checks of functional form.

## 5. Conclusions

Lower serum albumin was associated with recorded all-cause mortality after adjustment for clinical characteristics, NT-proBNP, and renal function. However, patients had different potential observation periods, which limits interpretation of the results. These findings do not establish causality or provide validated estimates of individual survival probability. Comparisons with PNI and HALP remain exploratory. External validation and follow-up with known event dates are needed before these models can be considered for clinical prediction.

## Figures and Tables

**Table 1:** Baseline characteristics by 2026 ESC heart failure phenotype. 2026 ESC phenotype definition: HFrEF = LVEF < 50%; HFpEF = LVEF ≥ 50%.

Characteristic	Overall (*N* = 492)	HFrEF (*N* = 215)	HFpEF (*N* = 277)	*p* Value
Demographics and cardiac phenotype				
Age, years	75 (67–81)	74 (65–80)	76 (68–82)	**0.037**
Male sex, *n* (%)	254 (51.6%)	137 (63.7%)	117 (42.2%)	**<0.001**
LVEF, %	50 (40–58)	38 (30–45)	55 (51–60)	**<0.001**
Laboratory characteristics				
NT-proBNP, pg/mL	4036 (1291–9500)	6737 (2612–13,054)	2500 (821–6276)	**<0.001**
CRP, mg/L	1.47 (0.62–3.67	1.28 (0.61–3.49)	1.54 (0.63–4.13)	0.548
Creatinine, µmol/L	100 (76–135)	104 (84–140)	97 (73–131)	**0.010**
High-sensitivity cardiac troponin I, pg/mL	15.6 (7.7–38.3)	23.4 (10.0–65.6)	12.7 (6.0–26.3)	**<0.001**
eGFR, mL/min/1.73 m^2^	54.3 (39.2–76.1)	53.8 (38.3–75.2)	55.4 (40.4–77.7)	0.331
Hemoglobin, g/L	128 (111–144)	130 (115–143)	127 (108–145)	0.141
Neutrophils, ×10^9^/L	6.27 (4.70–8.57)	6.17 (4.59–8.79)	6.30 (4.81–8.55)	0.581
Lymphocytes, ×10^9^/L	1.39 (0.96–1.94)	1.28 (0.94–1.91)	1.45 (0.96–1.96)	0.137
Platelets, ×10^9^/L	219 (176–274)	223 (178–272)	218 (175–278)	0.967
Albumin, g/L	39 (35–42)	38 (35–41)	39 (34–42)	0.254
PNI	45.9 (41.1–50.4)	45.6 (41.2–49.5)	46.4 (41.1–51.1)	0.131
HALP index	31.6 (17.9–49.5)	31.0 (18.4–43.1)	31.7 (17.3–53.6)	0.491
Comorbidities				
Coronary artery disease, *n* (%)	122 (24.8%)	60 (27.9%)	62 (22.4%)	0.159
Previous myocardial infarction, *n* (%)	69 (14.0%)	43 (20.0%)	26 (9.4%)	**<0.001**
Previous stroke, *n* (%)	72 (14.6%)	29 (13.5%)	43 (15.5%)	0.526
Diabetes mellitus, *n* (%)	166 (33.7%)	68 (31.6%)	98 (35.4%)	0.383
Hypertension, *n* (%)	396 (80.5%)	166 (77.2%)	230 (83.0%)	0.106
Obesity, *n* (%)	53 (10.8%)	23 (10.7%)	30 (10.8%)	0.962
Atrial fibrillation, *n* (%)	237 (48.2%)	114 (53.0%)	123 (44.4%)	0.058
Dyslipidemia, *n* (%)	188 (38.2%)	94 (43.7%)	94 (33.9%)	**0.027**
Pre-admission therapy				
Statin, *n* (%)	171 (34.8%)	78 (36.3%)	93 (33.6%)	0.532
ACE inhibitor, *n* (%)	201 (41.0%)	72 (33.6%)	129 (46.7%)	**0.003**
Beta-blocker, *n* (%)	342 (69.8%)	141 (65.9%)	201 (72.8%)	0.097
MRA, *n* (%)	163 (33.3%)	77 (36.0%)	86 (31.2%)	0.261
ARNI, *n* (%)	23 (4.7%)	18 (8.4%)	5 (1.8%)	**<0.001**
SGLT2 inhibitor, *n* (%)	146 (29.8%)	70 (32.7%)	76 (27.5%)	0.214
Diuretic, *n* (%)	288 (58.8%)	121 (56.5%)	167 (60.5%)	0.376

Notes. Continuous variables are median [IQR], and categorical variables are *n* (%). Comparisons used the Mann–Whitney U test and Pearson’s chi-square test, or Fisher’s exact test when required. Percentages use available-case denominators. NT-proBNP, albumin, LVEF, and statin use were available in all 492 patients; PNI/HALP and blood count variables in 491; CRP in 450; and high-sensitivity cardiac troponin I in 420. Each of the other therapy variables was available in 490 patients (HFrEF 214; HFpEF 276). Missing values were not imputed. LVEF defines the phenotype groups; its *p*-value is descriptive. Abbreviations: ACE, angiotensin-converting enzyme; ARNI, angiotensin receptor–neprilysin inhibitor; CRP, C-reactive protein; eGFR, estimated glomerular filtration rate; HFpEF, heart failure with preserved ejection fraction; HFrEF, heart failure with reduced ejection fraction; LVEF, left ventricular ejection fraction; MRA, mineralocorticoid receptor antagonist; NT-proBNP, N-terminal pro-B-type natriuretic peptide; SGLT2, sodium–glucose cotransporter 2. HALP, hemoglobin–albumin–lymphocyte–platelet index; PNI, prognostic nutritional index. Bold values indicate statistical significance (*p* < 0.05).

**Table 2:** Multivariable analysis of recorded all-cause mortality: clinical model.

Variable	Adjusted OR	95% CI	*p* Value
Age, per 10-year increase	1.32	1.08–1.60	0.005
Male sex	1.24	0.81–1.89	0.329
HFrEF (vs. HFpEF)	1.30	0.87–1.95	0.198
Coronary artery disease	0.84	0.49–1.44	0.533
Previous myocardial infarction	2.34	1.24–4.42	0.009
Previous stroke	1.47	0.86–2.53	0.161
Diabetes mellitus	0.98	0.64–1.51	0.942
Hypertension	0.65	0.40–1.06	0.085
Obesity	0.45	0.21–1.00	0.050
Atrial fibrillation	1.63	1.09–2.45	0.017
Dyslipidaemia	0.84	0.55–1.28	0.417

Model performance: *n* = 491; recorded deaths = 177; likelihood-ratio χ^2^ = 43.35 (df = 11), *p* < 0.001; AUC = 0.663; Brier score = 0.211. Notes. Odds ratios are mutually adjusted for all variables shown. Age is expressed per 10-year increase. Reference categories are female sex and HFpEF; for binary comorbidities, absence of the condition is the reference. No univariable selection was used. Missing outcome data were excluded; no imputation was performed. Abbreviations. AUC, area under the receiver operating characteristic curve; CI, confidence interval; HFpEF, heart failure with preserved ejection fraction; HFrEF, heart failure with reduced ejection fraction; OR, odds ratio.

**Table 3:** Multivariable analysis of recorded all-cause mortality: clinical and laboratory model.

Variable	Albumin Model		PNI Model		HALP Model	
	Adjusted OR (95% CI)	*p*	Adjusted OR (95% CI)	*p*	Adjusted OR (95% CI)	*p*
Age, per 10-year increase	1.24 (1.01–1.52)	0.039	1.21 (0.99–1.49)	0.065	1.24 (1.01–1.52)	0.040
Male sex	1.11 (0.71–1.73)	0.658	1.06 (0.68–1.66)	0.795	1.28 (0.82–1.98)	0.275
HFrEF (vs. HFpEF)	1.12 (0.72–1.73)	0.615	1.10 (0.71–1.71)	0.660	1.03 (0.67–1.58)	0.896
Coronary artery disease	0.88 (0.50–1.54)	0.653	0.87 (0.50–1.52)	0.637	0.85 (0.49–1.47)	0.556
Previous myocardial infarction	2.48 (1.27–4.83)	0.008	2.37 (1.22–4.61)	0.011	2.36 (1.22–4.57)	0.011
Previous stroke	1.41 (0.80–2.47)	0.237	1.47 (0.84–2.59)	0.179	1.59 (0.91–2.79)	0.106
Diabetes mellitus	0.98 (0.63–1.55)	0.944	0.99 (0.63–1.55)	0.955	0.95 (0.61–1.49)	0.835
Hypertension	0.71 (0.42–1.19)	0.191	0.72 (0.43–1.21)	0.220	0.71 (0.42–1.17)	0.179
Obesity	0.49 (0.21–1.11)	0.085	0.44 (0.19–1.01)	0.054	0.49 (0.22–1.11)	0.089
Atrial fibrillation	1.46 (0.95–2.25)	0.082	1.32 (0.86–2.03)	0.200	1.34 (0.88–2.05)	0.170
Dyslipidaemia	0.86 (0.55–1.34)	0.494	0.91 (0.58–1.42)	0.672	0.90 (0.58–1.39)	0.623
NT-proBNP, per doubling	1.13 (1.00–1.28)	0.051	1.13 (1.00–1.28)	0.051	1.19 (1.06–1.35)	0.004
eGFR, per 10 mL/min/1.73 m^2^ increase	0.97 (0.89–1.05)	0.417	0.97 (0.90–1.05)	0.508	1.00 (0.92–1.08)	0.974
Nutritional/inflammatory marker of interest						
Albumin, per 5 g/L decrease	1.60 (1.30–1.96)	**<0.001**	—	—	—	—
PNI, per 5-unit decrease	—	—	1.43 (1.21–1.67)	**<0.001**	—	—
HALP, per 10-unit decrease	—	—	—	—	1.15 (1.04–1.27)	**0.005**
Model performance						
Complete cases, *n*	491	—	490	—	490	—
Recorded deaths, *n*	177	—	177	—	177	—
AUC	0.735	—	0.732	—	0.710	—
Brier score	0.194	—	0.195	—	0.201	—
Overall model LR χ^2^ (df = 14)	79.22	<0.001	77.55	<0.001	66.24	<0.001
Incremental LR χ^2^ for marker (df = 1)	21.10	<0.001	20.07	<0.001	8.77	0.003

Values are adjusted odds ratios (OR) with 95% confidence intervals. Each model included age, sex, 2026 ESC HF phenotype, coronary artery disease, previous myocardial infarction, previous stroke, diabetes mellitus, hypertension, obesity, atrial fibrillation, dyslipidaemia, log_2_-transformed NT-proBNP, eGFR, and one nutritional/inflammatory marker (albumin, PNI, or HALP). PNI = albumin (g/L) + 5 × lymphocyte count (10^9^/L). HALP = hemoglobin (g/L) × albumin (g/L) × lymphocyte count (10^9^/L)/platelet count (10^9^/L). Incremental LR tests compare each marker-containing model with the same background model without that marker. Bold values indicate statistical significance (*p* < 0.05). Abbreviations. AUC: area under the receiver operating characteristic curve; LR: Likelihood ratio; df: Degrees of freedom; eGFR: Estimated glomerular filtration rate; NT-proBNP: N-terminal pro-B-type natriuretic peptide; PNI: Prognostic nutritional index; HALP: Hemoglobin–albumin–lymphocyte–platelet index; CI, confidence interval; HFpEF, heart failure with preserved ejection fraction; HFrEF, heart failure with reduced ejection fraction.

**Table 4:** Incremental discrimination of sequential models for recorded all-cause mortality.

Model	AUC (95% CI)	ΔAUC (95% CI)	LR χ^2^ (*p*)	Brier Score
Clinical model	0.662 (0.613–0.712)	—	—	0.212
Clinical + NT-proBNP	0.689 (0.641–0.738)	+0.027 (−0.001–0.055)	14.64 (<0.001)	0.205
Clinical + NT-proBNP + albumin	0.731 (0.684–0.777)	+0.042 (0.012–0.071)	20.35 (<0.001)	0.195
Clinical + NT-proBNP + PNI	0.730 (0.683–0.776)	+0.041 (0.012–0.069)	19.66 (<0.001)	0.195
Clinical + NT-proBNP + HALP	0.710 (0.663–0.758)	+0.021 (0.001–0.041)	8.79 (0.003)	0.201

All models used the same 490 complete cases, including 177 deaths. The clinical model included age, sex, HF phenotype, coronary artery disease, previous myocardial infarction, stroke, diabetes, hypertension, obesity, atrial fibrillation, and dyslipidemia. NT-proBNP was log_2_-transformed. Markers were added separately; eGFR was not included. ΔAUC and LR tests compare Clinical + NT-proBNP with Clinical, and each marker model with Clinical + NT-proBNP. ΔAUC confidence intervals use the DeLong covariance estimate and describe apparent performance. Abbreviations. AUC: area under the receiver operating characteristic curve; CI, confidence interval; LR: Likelihood ratio; eGFR: Estimated glomerular filtration rate; NT-proBNP: N-terminal pro-B-type natriuretic peptide; PNI: Prognostic nutritional index; HALP: Hemoglobin–albumin–lymphocyte–platelet index; HF: Heart failure.

## Data Availability

The datasets used and analyzed during the current study are available from the corresponding author on reasonable request.

## Supplementary Materials

The following supporting information can be downloaded at: https://www.mdpi.com/article/10.3390/jcm15218231/s1. Table S1. Calibration and internal validation of the primary albumin model for recorded all-cause mortality; Table S2. Multicollinearity in the albumin, PNI, and HALP models; Table S3. Associations of albumin, PNI, and HALP with recorded all-cause mortality by LVEF phenotype; Table S4. Sensitivity analysis in patients with at least 12 months of potential observation; Table S5. Restricted cubic spline analysis of serum albumin and recorded all-cause mortality; Table S6. Exploratory sensitivity analysis of recorded all-cause mortality with additional adjustment for C-reactive protein. Figure S1. Serum albumin and recorded all-cause mortality. Figure S2. Flow diagram of patient selection.

## Abbreviations

The following abbreviations are used in this manuscript:

ACE	Angiotensin-converting enzyme
AF	Atrial fibrillation
ARNI	Angiotensin receptor–neprilysin inhibitor
AUC	Area under the receiver operating characteristic curve
BMI	Body mass index
CAD	Coronary artery disease
CAR	C-reactive protein-to-albumin ratio
CI	Confidence interval
CRP	C-reactive protein
df	Degrees of freedom
eGFR	Estimated glomerular filtration rate
EF	Ejection fraction
ESC	European Society of Cardiology
HALP	Hemoglobin–albumin–lymphocyte–platelet index
HF	Heart failure
HFpEF	Heart failure with preserved ejection fraction
HFrEF	Heart failure with reduced ejection fraction
IQR	Interquartile range
LR	Likelihood ratio
LVEF	Left ventricular ejection fraction
MDRD	Modification of Diet in Renal Disease
MI	Myocardial infarction
MIMIC-IV	Medical Information Mart for Intensive Care IV
MRA	Mineralocorticoid receptor antagonist
NT-proBNP	N-terminal pro-B-type natriuretic peptide
OR	Odds ratio
PNI	Prognostic nutritional index
ROC	Receiver operating characteristic
SGLT2	Sodium-glucose cotransporter 2
VIF	Variance inflation factor
