# Supplementary material for: Serum Albumin and Albumin-Based Nutritional-Inflammatory Indices Across the 2026 ESC Heart Failure Phenotypes: Associations with Recorded All-Cause Mortality
Source: J Clin Med. 2026 Oct 22;15(21):8231. doi: 10.3390/jcm15218231 (PMC13764357; doi:10.3390/jcm15218231)
Supplement: Supplementary file 1 [file jcm-15-08231-s001.zip › jcm-4640725-Supplementary Figure S1.pdf]

Supplementary Figure S1. Serum albumin and recorded all-cause mortality

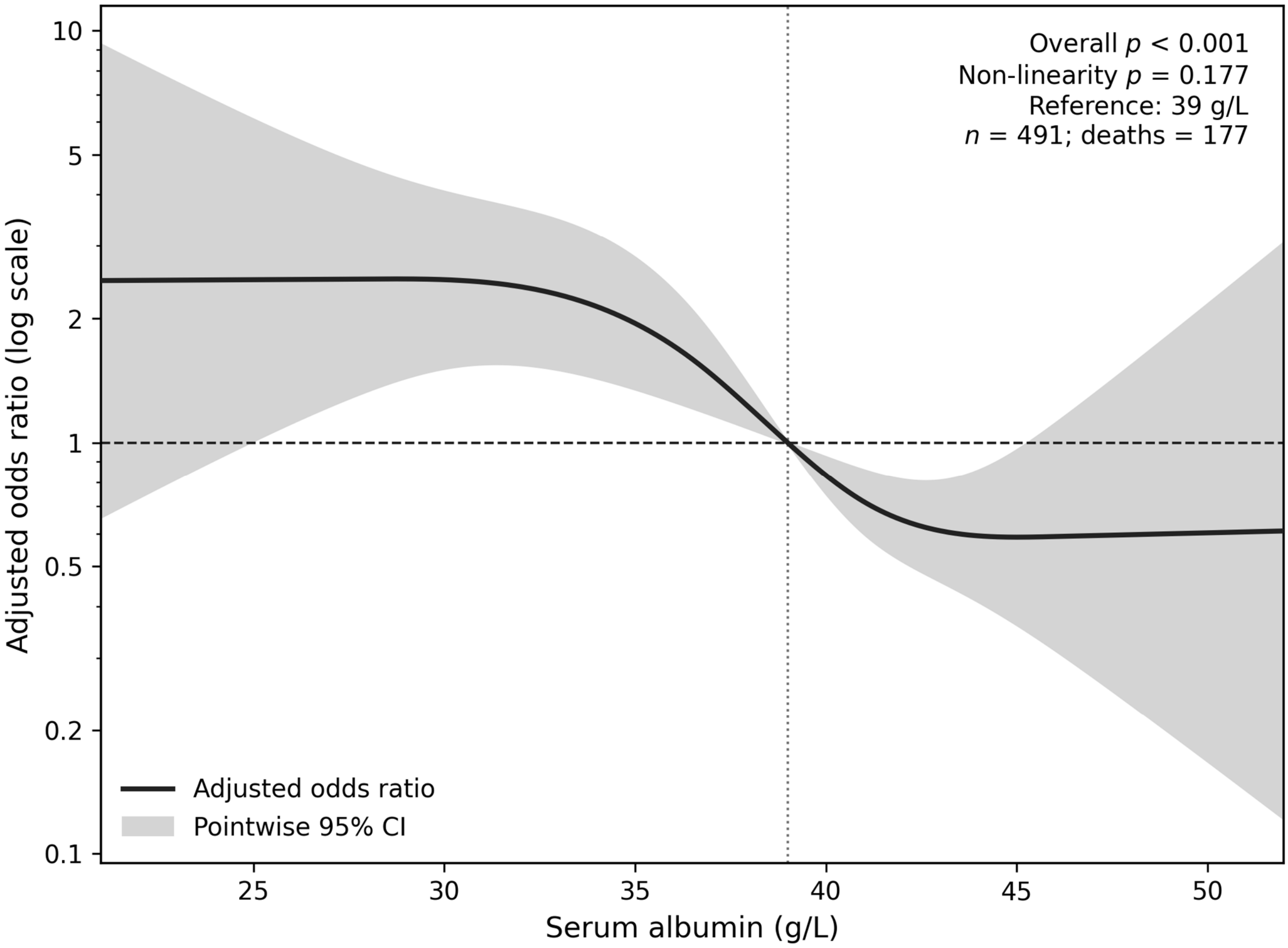

Adjusted association between serum albumin and recorded all-cause mortality, estimated using the multivariable restricted cubic spline logistic regression model described in Table S5 (491 complete cases; 177 recorded deaths). The solid line shows odds ratios relative to 39.0 g/L; the shaded area shows pointwise 95% confidence intervals. Four knots were placed at 28.0, 36.5, 41.0, and 46.0 g/L. The horizontal dashed line marks an odds ratio of 1 and the vertical dotted line marks the reference concentration. The odds ratio axis uses a logarithmic scale. Likelihood-ratio tests: overall association, $p < 0.001$; non-linearity, $p = 0.177$.
