# Supplementary material for: Serum Albumin and Albumin-Based Nutritional-Inflammatory Indices Across the 2026 ESC Heart Failure Phenotypes: Associations with Recorded All-Cause Mortality
Source: J Clin Med. 2026 Oct 22;15(21):8231. doi: 10.3390/jcm15218231 (PMC13764357; doi:10.3390/jcm15218231)
Supplement: Supplementary file 1 [file jcm-15-08231-s001.zip › jcm-4640725-Supplementary Table S1.pdf]

Supplementary Table S1. Calibration and internal validation of the primary albumin model for recorded all-cause mortality

Metric	Value
Complete cases, n	491
Recorded deaths, n	177
Apparent AUC	0.735
Apparent Brier score	0.194
Hosmer-Lemeshow χ^2 (df=8)	11.67
Hosmer-Lemeshow p value	0.167
Valid bootstrap resamples / attempts	1000/1001
Mean AUC optimism	0.033
Optimism-corrected AUC	0.702
Optimism-corrected Brier score	0.207
Optimism-corrected calibration slope	0.822
Optimism-corrected calibration intercept	-0.089

Model. Of 492 patients with available serum albumin, LVEF, and NT-proBNP, 491 had known mortality status and complete model data. The covariates were age, sex, 2026 ESC HF phenotype, coronary artery disease, previous myocardial infarction, previous stroke, diabetes mellitus, arterial hypertension, obesity, atrial fibrillation, dyslipidaemia, $\log_2$-transformed NT-proBNP, eGFR, and serum albumin. Albumin was modelled per 5 g/L decrease and eGFR per 10 mL/min/1.73 m^2 increase.

Bootstrap validation. There were 1000 valid bootstrap samples from 1001 draws (random seed 20260929). One draw was excluded because of quasi-complete separation. The model was refitted in each sample and evaluated in that sample and the original cohort. Optimism was the bootstrap estimate minus the original-cohort estimate for the same refitted model. Mean optimism was subtracted from the apparent value. Calibration intercepts and slopes were estimated by logistic regression of mortality status on the model linear predictor. Validation was limited to the primary albumin model; the PNI and HALP models were not bootstrap-validated.

The decrease in AUC after correction and a calibration slope below 1 suggest some overfitting.
