# Supplementary material for: Serum Albumin and Albumin-Based Nutritional-Inflammatory Indices Across the 2026 ESC Heart Failure Phenotypes: Associations with Recorded All-Cause Mortality
Source: J Clin Med. 2026 Oct 22;15(21):8231. doi: 10.3390/jcm15218231 (PMC13764357; doi:10.3390/jcm15218231)
Supplement: Supplementary file 1 [file jcm-15-08231-s001.zip › jcm-4640725-Supplementary Table S2.pdf]

Supplementary Table S2. Multicollinearity in the albumin, PNI, and HALP models

Variable	Albumin model		PNI model		HALP model	
	Tolerance	VIF	Tolerance	VIF	Tolerance	VIF
Age	0.734	1.363	0.726	1.377	0.731	1.367
Male sex	0.813	1.230	0.806	1.240	0.825	1.212
HFrEF (vs. HFpEF)	0.824	1.214	0.825	1.212	0.830	1.205
Coronary artery disease	0.699	1.431	0.700	1.430	0.698	1.432
Previous myocardial infarction	0.729	1.373	0.730	1.370	0.727	1.375
Previous stroke	0.950	1.053	0.952	1.050	0.946	1.057
Diabetes mellitus	0.898	1.114	0.897	1.115	0.895	1.117
Hypertension	0.913	1.095	0.910	1.099	0.911	1.098
Obesity	0.880	1.137	0.881	1.135	0.883	1.132
Atrial fibrillation	0.852	1.173	0.847	1.180	0.849	1.177
Dyslipidaemia	0.871	1.148	0.867	1.153	0.866	1.154
$\log_2$-transformed NT-proBNP	0.650	1.539	0.649	1.540	0.703	1.422
eGFR	0.761	1.314	0.771	1.297	0.795	1.258
**Albumin**	**0.831**	**1.204**	—	—	—	—
**PNI**	—	—	**0.791**	**1.265**	—	—
**HALP**	—	—	—	—	**0.888**	**1.126**

VIF, variance inflation factor. The included study cohort comprised 492 patients with available serum albumin, LVEF, and NT-proBNP. Analyses used the complete-case samples for the albumin (n=491), PNI (n=490), and HALP (n=490) models. Each model included the covariates shown in the table and one of the three markers. HF phenotype followed the 2026 ESC classification. The highest VIF was 1.540 and the lowest tolerance was 0.649.
