# Supplementary material for: Serum Albumin and Albumin-Based Nutritional-Inflammatory Indices Across the 2026 ESC Heart Failure Phenotypes: Associations with Recorded All-Cause Mortality
Source: J Clin Med. 2026 Oct 22;15(21):8231. doi: 10.3390/jcm15218231 (PMC13764357; doi:10.3390/jcm15218231)
Supplement: Supplementary file 1 [file jcm-15-08231-s001.zip › jcm-4640725-Supplementary Table S3.pdf]

Supplementary Table S3. Associations of albumin, PNI, and HALP with recorded all-cause mortality by LVEF phenotype

Marker	HFpEF Adjusted OR (95% CI)	HFrEF Adjusted OR (95% CI)	p for interaction
Albumin, per 5 g/L decrease	1.44 (1.11–1.87)	1.87 (1.35–2.59)	0.212
PNI, per 5-unit decrease	1.30 (1.06–1.59)	1.63 (1.26–2.10)	0.166
HALP, per 10-unit decrease	1.10 (0.97–1.25)	1.23 (1.05–1.45)	0.275

Values are adjusted odds ratios (OR) with 95% confidence intervals for recorded all-cause mortality within each 2026 ESC LVEF phenotype. Models included age, sex, HF phenotype, coronary artery disease, previous myocardial infarction, previous stroke, diabetes mellitus, hypertension, obesity, atrial fibrillation, dyslipidaemia, $\log_2$-transformed NT-proBNP, eGFR, one marker, and its interaction with HF phenotype. Interaction p values were obtained from likelihood-ratio tests comparing models with and without the interaction term. The study cohort comprised 492 patients with available serum albumin, LVEF, and NT-proBNP. The albumin analysis included 491 patients (HFpEF 276, HFrEF 215); PNI and HALP analyses included 490 each (HFpEF 275, HFrEF 215), with 177 recorded deaths in each analysis. ORs were numerically higher in HFrEF, but the interaction tests did not show statistically significant differences by phenotype. PNI = albumin (g/L) + 5 × lymphocyte count (10^9/L). HALP = haemoglobin (g/L) × albumin (g/L) × lymphocyte count (10^9/L) / platelet count (10^9/L).
