# Supplementary material for: Serum Albumin and Albumin-Based Nutritional-Inflammatory Indices Across the 2026 ESC Heart Failure Phenotypes: Associations with Recorded All-Cause Mortality
Source: J Clin Med. 2026 Oct 22;15(21):8231. doi: 10.3390/jcm15218231 (PMC13764357; doi:10.3390/jcm15218231)
Supplement: Supplementary file 1 [file jcm-15-08231-s001.zip › jcm-4640725-Supplementary Table S4.pdf]

Supplementary Table S4. Sensitivity analysis in patients with at least 12 months of potential observation

Marker	Primary analysis Adjusted OR (95% CI)	≥12-month sensitivity analysis Adjusted OR (95% CI)	Marker association p value	Sensitivity AUC	Incremental LR p
Albumin, per 5 g/L decrease	1.60 (1.30–1.96)	1.66 (1.34–2.04)	<0.001	0.741	<0.001
PNI, per 5-unit decrease	1.43 (1.21–1.67)	1.45 (1.23–1.70)	<0.001	0.735	<0.001
HALP, per 10-unit decrease	1.15 (1.04–1.27)	1.15 (1.05–1.27)	0.004	0.710	0.003

All 482 patients who met the date criterion had known mortality status and complete data for the albumin model, with 173 recorded deaths. One patient lacked the blood count data needed for PNI and HALP, leaving 481 complete cases and 173 recorded deaths in each model.

Mortality status was ascertained on 17 September 2026. All 492 patients in the final cohort had available serum albumin, LVEF, and NT-proBNP, as well as an index hospitalization date. The sensitivity analysis included those admitted on or before 17 September 2025, allowing at least 12 months of potential observation.

Models included age, sex, 2026 ESC HF phenotype, coronary artery disease, previous myocardial infarction, previous stroke, diabetes mellitus, arterial hypertension, obesity, atrial fibrillation, dyslipidaemia, $\log_2$-transformed NT-proBNP, eGFR, and one marker. Each model used complete cases. Incremental likelihood-ratio tests compared models with and without the marker in the same patients. The marker p value is the two-sided Wald p value in the sensitivity model. AUC and incremental LR p values also refer to the sensitivity models.

Primary estimates are shown for comparison; no statistical test compared the primary and sensitivity ORs. Restricting the cohort by admission date ensures at least 12 months of potential observation, but does not equalize observation time or replace time-to-event analysis.

Abbreviations: AUC, area under the receiver operating characteristic curve; CI, confidence interval; eGFR, estimated glomerular filtration rate; HALP, haemoglobin-albumin-lymphocyte-platelet index; HF, heart failure; LR, likelihood ratio; NT-proBNP, N-terminal pro-B-type natriuretic peptide; OR, odds ratio; PNI, prognostic nutritional index.
