# Supplementary material for: Serum Albumin and Albumin-Based Nutritional-Inflammatory Indices Across the 2026 ESC Heart Failure Phenotypes: Associations with Recorded All-Cause Mortality
Source: J Clin Med. 2026 Oct 22;15(21):8231. doi: 10.3390/jcm15218231 (PMC13764357; doi:10.3390/jcm15218231)
Supplement: Supplementary file 1 [file jcm-15-08231-s001.zip › jcm-4640725-Supplementary Table S5.pdf]

Supplementary Table S5. Restricted cubic spline analysis of serum albumin and recorded all-cause mortality

Analysis	LR χ^2	df	p value	AUC	Brier score
Overall association of albumin (spline vs. model without albumin)	24.56	3	<0.001	0.739	0.193
Non-linearity (spline vs. linear albumin model)	3.46	2	0.177	—	—

The analysis used 491 complete cases with 177 recorded deaths from a cohort of 492 patients with available serum albumin, LVEF, and NT-proBNP. One patient was excluded because mortality status was missing.

Four knots were placed at 28.0, 36.5, 41.0, and 46.0 g/L (5th, 35th, 65th, and 95th percentiles of the albumin distribution in the 491 complete cases). The reference was the median albumin concentration of 39.0 g/L.

The model included age, sex, 2026 ESC HF phenotype, coronary artery disease, previous myocardial infarction, previous stroke, diabetes mellitus, arterial hypertension, obesity, atrial fibrillation, dyslipidaemia, $\log_2$-transformed NT-proBNP, eGFR, and serum albumin entered as a restricted cubic spline.

Likelihood-ratio tests compared the spline model with the model without albumin (overall association) and with the model containing a linear albumin term (non-linearity). There was no evidence of significant non-linearity. The linear model had an AUC of 0.735 and a Brier score of 0.194. Supplementary Figure S1 shows the fitted curve and pointwise 95% confidence limits.

Abbreviations: AUC, area under the receiver operating characteristic curve; eGFR, estimated glomerular filtration rate; HF, heart failure; LR, likelihood ratio; NT-proBNP, N-terminal pro-B-type natriuretic peptide.
