# Supplementary material for: Serum Albumin and Albumin-Based Nutritional-Inflammatory Indices Across the 2026 ESC Heart Failure Phenotypes: Associations with Recorded All-Cause Mortality
Source: J Clin Med. 2026 Oct 22;15(21):8231. doi: 10.3390/jcm15218231 (PMC13764357; doi:10.3390/jcm15218231)
Supplement: Supplementary file 1 [file jcm-15-08231-s001.zip › jcm-4640725-Supplementary Table S6.pdf]

Supplementary Table S6

Exploratory sensitivity analysis of recorded all-cause mortality with additional adjustment for C-reactive protein

A Marker associations

Marker and scale	n / deaths	Without CRP OR (95% CI)	p	With CRP OR (95% CI)	p
Albumin per 5 g/L decrease	449 / 169	1.693 (1.363–2.103)	<0.001	1.577 (1.257–1.978)	<0.001
PNI per 5-unit decrease	448 / 169	1.456 (1.233–1.719)	<0.001	1.372 (1.151–1.635)	<0.001
HALP per 10-unit decrease	448 / 169	1.140 (1.032–1.260)	0.010	1.100 (0.994–1.218)	0.066

B CRP associations

Model	CRP per doubling OR (95% CI)	p
Albumin model	1.130 (0.998–1.279)	0.054
PNI model	1.132 (0.999–1.281)	0.051
HALP model	1.189 (1.054–1.340)	0.005

Notes. Models included age, sex, HF phenotype (LVEF <50% vs ≥50%), coronary artery disease, previous myocardial infarction, previous stroke, diabetes mellitus, hypertension, obesity, atrial fibrillation, dyslipidaemia, $\log_2$-transformed NT-proBNP, eGFR and one marker. CRP was $\log_2$-transformed.

Models with and without CRP used the same complete cases for each marker. CRP was missing in 42 of 492 patients (8.5%). p values are from two-sided Wald tests.

Abbreviations. CI, confidence interval; CRP, C-reactive protein; eGFR, estimated glomerular filtration rate; HALP, haemoglobin-albumin-lymphocyte-platelet index; HF, heart failure; LVEF, left ventricular ejection fraction; NT-proBNP, N-terminal pro-B-type natriuretic peptide; OR, odds ratio; PNI, prognostic nutritional index.
